# The influence of motivation, self-efficacy, and fear of failure on the career adaptability of vocational school students: Moderated by meaning in life

**DOI:** 10.3389/fpsyg.2022.958334

**Published:** 2022-09-21

**Authors:** Ya-Ting Chuang, Tzu-Huang Huang, Shin-Yi Lin, Bo-Ching Chen

**Affiliations:** ^1^Department of Education Curriculum and Instruction, National University of Tainan, Tainan, Taiwan; ^2^Academic Affairs Office, National Nanke International Experimental High School, Tainan, Taiwan; ^3^Department of Education, Educational Entrepreneurship and Management, National University of Tainan, Tainan, Taiwan; ^4^Physical Education Research and Development Center, National Taiwan Normal University, Taipei City, Taiwan

**Keywords:** career construction theory, self-determination theory, career adaptability, task motivation, life meaning, PISA

## Abstract

It is an important issue for vocational school students to have good adaptability for their future life. This study combines career construction theory and self-determination theory to construct a model to explore the relationship between the “motivation,” “self-efficacy,” “fear of failure,” “career adaptability,” and “meaning in life” of vocational school students. This study used a secondary data research method and retrieved a total of 2,377 data from vocational school students in Taiwan from the perspective of data exploration using PISA 2018 data, which was validated by the partial least squares structural equation model (PLS-SEM). The following results were obtained: (1) Vocational students were afraid that failure would have a negative impact on their career adaptability. (2) Motivation and Self-efficacy had a positive effect on career adaptability. (3) Motivation positively affected fear of failure. (4) Self-efficacy negatively affected fear of failure. (5) Meaning in life could positively moderate the effect of self-efficacy on fear of failure. (6) However, there was no statistical difference in the moderating effect of meaning in life on the relationship between motivation and fear of failure. First, fear of failure negatively affected career adaptability, while motivation and self-efficacy positively affected career adaptability; compared to the three effects, the negative effect of fear of failure may not be as great as expected. Second, motivation is like a double-edged sword as it improves adaptability, but it also comes with an increased fear of failure. On the contrary, self-efficacy can simultaneously improve the career adaptability of vocational students and reduce their fear of failure. Therefore, the development of self-efficacy should be given priority over motivation in the career adaptability enhancement strategy of vocational students. Finally, the meaning of life can positively moderate the negative influence of self-efficacy on the fear of failure. In other words, for vocational students with a low sense of self-efficacy, perhaps life education can be used instead as a strategy to reduce their fear of failure.

## Introduction

Since 2010, interest in career adaptability (CA) has been growing, and numerous empirical studies have found that CA and self-efficacy (SE) are considered to be important resources in the career decision-making process that can successfully influence career development in an uncertain work environment (Porfeli and Savickas, 2012; Duffy et al., 2015; Rudolph et al., 2017a; Stead et al., 2021). Especially in the face of today’s rapidly changing society, the occupational landscape has become more diverse, boundaryless, non-linear, fragmented, and global than ever imagined (Jiang, 2017). Having a high level of CA will prepare vocational students for the uncertainty of future career tasks, career roles, and work environments (Savickas, 1997; Feng et al., 2021). When vocational students have good CA, it increases their chances of finding high-quality jobs after graduation (Koen et al., 2012; Guan et al., 2013; Feng et al., 2021). Therefore, the transition period from student life to professional career is very important for vocational students (Koen et al., 2012). In other words, at this stage, it is necessary for vocational students to enhance their CA in order to meet the needs of the future job market, so many scholars are now concerned about the CA of student groups. (Lazarová et al., 2019; Zhang et al., 2021; Kenny et al., 2022).

Savickas’s (1997) career construction theory (CCT) is a constructivist view of CA as a psychosocial construct that can manage one’s career and the resources needed to change careers (Savickas, 1997, 2005). CA consists of four dimensions: career concern, career control, career curiosity, and career confidence (Savickas, 1997, 2005). Through these four dimensions, we explored how people can enhance their abilities and explore their career options and opportunities, and navigate through the transition or unemployment period (Koen et al., 2012). In other words, CA is the core concept of the entire career construct theory.

Many studies have been conducted in the past to demonstrate the impact of motivation (MT) on CA (Fang et al., 2018; Schuesslbauer et al., 2018). From the self-determination theory (SDT) point of view, people have autonomy needs, competence needs, and relationship needs, and these three different types of needs all have an impact on intrinsic motivation (Deci and Ryan, 2012; Ryan and Deci, 2017; Vasconcellos et al., 2020; Howard et al., 2021). When needs are met, people are motivated to accomplish specific tasks on their own. In contrast, when these needs are not met or are only partially met, individuals will change their behavior due to lack of motivation (McDavid et al., 2014; Mouratidis et al., 2015; Howard et al., 2021). In other words, there seems to be a certain degree of correlation between psychological needs, MT, and CA (Ryan and Deci, 2017; van Aart et al., 2017; Howard et al., 2021).

Based on the above two theoretical perspectives of CCT and SDT, first of all, it can be confirmed that the most critical point of career development of vocational students is CA, and the process of constructing CA includes career concern, career control, career curiosity, and career confidence influence (Savickas, 1997, 2005). Since vocational students’ career development will be affected by different degrees of willingness and ability (Rudolph et al., 2017b). Motivation is the source of willingness and ability; therefore, motivation is one of the most important factors affecting the behavior of vocational students.

Secondly, because of the SDT competency requirement, which is the need to be able to effectively master the environment and to feel success and growth in it, the competency requirement is equivalent to SE (Sweet et al., 2012; Adams et al., 2017; Ryan and Deci, 2017; Guay et al., 2020). Conversely, when people feel that they cannot effectively control their environment, they develop a negative tendency to try to avoid the pressure to fail (fear of failure) (Weinstein and Ryan, 2011; Krijgsman et al., 2017). This shows that there is a relationship between SE, fear of failure (FF), and the CA of vocational students.

Finally, CA is what enables people to expand and refine their self-concept in their professional roles and to create meaning in their lives from it (Savickas, 2005; Koen et al., 2012). The meaning of life (ML) is a subjective experience that people realize in their daily life that they think has value, and this experience can stimulate people’s drive to achieve their goals in the long term, and can make them passionate and willing to keep investing time and effort until they achieve their desired goals (Steger et al., 2006; Li et al., 2018; Kıymaz, 2019). In other words, there is an interaction between ML and CA, and it will deeply affect the future career development of vocational students (Praskova et al., 2014; Buyukgoze-Kavas et al., 2015; Yuen and Yau, 2015).

Regarding CA, past studies have focused on general company employees (AlKhemeiri et al., 2020; Omar and Tajudeen, 2020; Wang et al., 2021), student veterans or veterans (Ghosh and Fouad, 2018; Ghosh et al., 2019; Becker et al., 2022), college students (Gregor et al., 2021; Hu et al., 2021; Kim and Smith, 2021), high school students (Dostanić et al., 2021), adolescent groups (Liang et al., 2020; Bacanli and Sarsikoğlu, 2021), and working women (Takawira, 2020), and have revealed that the target population is very diverse and represents the importance of research on CA.

However, further studies were found in various professional fields with samples including nursing students (Tian and Fan, 2014; İspir et al., 2019; Zhang et al., 2022), hospitality and tourism students (Ramaprasad et al., 2022), student athletes (Nikander et al., 2022), and so on. All of the above studies were conducted with a specific target population at the beginning of the study. Few studies have been conducted to collect data on a wide range of vocational students, and so it is not possible to understand the overall situation of career adaptability of general vocational students.

In the past, most of these studies focused on students’ parental behaviors, expectations of students, and caring support (Liang et al., 2020; Parola and Marcionetti, 2021; Nikander et al., 2022). External variables, or students’ internal personality traits and active personality (Bacanli and Sarsikoğlu, 2021; Hu et al., 2021; Chang and Liu, 2022) have been less frequently explored in terms of MT, SE, and other intrinsic subjective drivers that are at the core of a person’s being from a self-deterministic perspective.

Based on the above background motives, many studies on CA have been carried out, but there are few empirical studies on enhancing students’ CA, especially when vocational students who have just entered vocational school at the age of 16, have just gone through the career transition process from junior high school students to vocational students. Therefore, the purpose of this study was to investigate the relationship between humanistic MT, SE, FF, and CA of vocational students.

### Career adaptability

The quest for global sustainability and technological demands requires a shift in our culture to embrace a new vision. Adaptability refers to a person’s awareness that in any given situation there are alternatives, including a willingness to be flexible and adaptable to the different situation (Martin and Rubin, 1995). In any given situation, one can choose how to behave. Students’ CA effectively manages their responses to change, and novelty and uncertainty are critical to managing and meeting academic demands. CA is considered particularly relevant to students because the classroom is a dynamic environment that is constantly changing (Burns et al., 2018).

Before deciding to adjust their behavior, students’ go through a social cognitive process during which they become aware of alternative ways of doing things. Students who are able to identify possible adjustments based on situational factors are cognitively more flexible than those who see only one correct behavioral response (Roloff and Berger, 1982; Lippard-Justice, 1989). CA can be conceptually distinguished from other mental constructs that focus on successfully overcoming adversity, such as buoyancy, resilience, and mental toughness (Putwain et al., 2019).

### Fear of failure

Fear of failure is the tendency to evaluate threats in an evaluation situation where failure is possible. This motivation is socialized from early childhood and is rooted in a tendency to self-evaluation. FF is a tendency to try to avoid failure due to the expectation of shame, humiliation, or embarrassment when completing a performance task. FF is not only a product of the perceived learning environment, but can also come from internal sources, and students’ performance goal orientation motivation is particularly closely linked to FF (Sagar and Lavallee, 2010; Giel et al., 2020; Taylor et al., 2021).

FF avoidance MT drives the adoption of avoidance goals, which can have negative effects on individuals, including lower levels of well-being and lower intrinsic MT (Elliot and Harackiewicz, 1996; Elliot and Church, 1997; Conroy and Elliot, 2004). In education, FF sparks an assessment of the negative consequences (e.g., scoffing, shame, or nervousness in front of the class) on the students’ well-being of those potential emotions that students may feel after not performing a task correctly in front of their peers and teachers (Manuel et al., 2020) and increases their anxiety and depression, poor performance, and dropout rates (Sagar and Lavallee, 2010).

### Motivation

Motivation is a term used to explain behavior; it generally refers to what makes us act and leads us to purposeful behavior. More specifically, MT has been defined as behavior directed toward a result or goal, where the intensity of the behavior or intensity of engagement may vary (Wigfield and Eccles, 2000; Deci and Ryan, 2002; Reeve, 2014). This dimension of motivational qualities is driven by the satisfaction or frustration of innate basic psychological needs: competence, autonomy, and kinship. The more the environment meets these needs, the more spontaneous forms of MT will emerge, and the individual will experience a higher level of well-being. The more active students are, the more time they spend studying (Deci and Ryan, 1987; Lens and Vansteenkiste, 2020). MT is an important factor in determining levels of athletic, work, and academic performance. In many tasks, MT affects performance levels, the efficiency with which tasks are used, and the transfer of competence through the use of time. MT is related to the psychological factors that drive behavior and choice, and intrinsic motivation and previous learning attitudes can be precursors to task engagement (Brooks et al., 2012; Davis and McPartland, 2012). The combination of strong MT and high task commitment promotes a successful learning experience.

### Self-efficacy

Self-efficacy is the belief in one’s ability to influence events that affect one’s life and control over the way these events are experienced. Also, it refers to the belief in one’s capabilities to organize and execute the courses of action required to manage prospective situations (Bandura, 1994, 1997), as well as one’s sense of self, personal perceptions, beliefs, judgments, and feelings about who one is as a person. Many psychologists distinguish between self-concept assessment of one’s own characteristics, strengths, weaknesses, and self-esteem judgments, and feelings about one’s own value and worth which are two aspects of the sense of self (Ormrod, 2009). Those two aspects closely overlap; however, on the whole these two terms are often used interchangeably. SE is about the degree to which one can succeed in certain activities and accomplish certain goals. SE is grounded in the theoretical framework of social cognitive theory which emphasizes the evolution and exercise of human agency that allows people to exercise some influence over what they do (Bandura, 2006). Vocational students’ specific SE for various tasks and activities contribute to their more general sense of self. SE is more focused on the feeling that vocational students have and are up for challenges.

### Meaning in life

The meaning of a person’s life cannot be inferred just by knowing his or her objective situation. Life meaning means an understandable feeling which is a very subjective thing, a quality that is universal in one’s inner life. When a person commits to a goal and has a purpose, a sense of significance and value in life may raise one’s ML (George and Park, 2013). When asking about the meaning of someone’s life, we are asking about the quality of his or her inner thoughts and emotional experience (Klinger, 1977). Whereas goals refer to a sense of core purpose and direction in life, meaning is about the intrinsic worth of life and the feeling of having a life worth living (Martela and Steger, 2016). A meaningful life is illustrational as a better, rarer, and inspiring life than a happy life (Ward and King, 2016). Considering that socioeconomic status is positively associated with ML, as this association encounters assumptions about ML beyond life quotas, researchers typically treat socioeconomic status as a control variable (Kobau et al., 2010). Also, socioeconomic status is as much a source of ML as religious beliefs and social relationships (Ward and King, 2016).

To understand this experience, we must listen without affecting what the data tell us about this subjective state, even as they challenge our assumptions and enhance the ML through its benefits (Heintzelman and King, 2014). The fact of ML as a subjective experience posits that vocational students’ experience what they perceive as meaning in life to varying degrees and that they can report on that experience.

## Research model and hypotheses

### Research hypotheses

#### The relationship between MT, SE, FF, and CA

Fear of failure is seen as a tendency to avoid failure in the environment in which one grows up (Conroy and Elliot, 2004; Alabduljabbar et al., 2022). Vocational students with this tendency may experience negative psychological effects such as extreme shame, embarrassment, low self-esteem, fear of upsetting significant others, and so on when they encounter failure (Conroy and Elliot, 2004; McGregor and Elliot, 2005; Şeker, 2019; Vaughn et al., 2021; Alabduljabbar et al., 2022). In order to avoid the embarrassment of failure, they often use self-imposed limits and other means to protect themselves from the consequences of failure (Elliot and Thrash, 2004; Vaughn et al., 2021). This shows that the higher the fear of failure, the less effort they will put into their career goals, thus reducing their psychological feelings when they fail, resulting in lower CA.

Many studies have been conducted in the past to confirm the impact of MT on CA (Fang et al., 2018; Schuesslbauer et al., 2018). MT can raise the level of importance that vocational students attach to their careers (Fuller and MacFadyen, 2012). It is also an important factor that affects students’ participation and academic performance in the classroom (Nayir, 2017). It allows vocational students to be more motivated to improve their abilities to face challenges (such as participating in English competitions) or to pass relevant vocational certification examinations (Kudo et al., 2013; Liu, 2020). This will help vocational students find jobs more easily after graduation (Sarımehmet et al., 2021). In other words, the higher the MT of vocational students, the more they will pay attention to the future development of related skills and the higher their CA.

SE is a person’s belief that he or she is capable of accomplishing a certain behavior (Bandura, 1994, 1997). Vocational students with SE will plan and predict their future career vision in advance, adjust their behavior, and have more confidence when facing obstacles (Miraglia et al., 2015; Bubic, 2017; Hou et al., 2019). Vocational students with a high level of SE have more confidence to overcome all difficulties when facing unknown and unpredictable professional environments. Therefore, when vocational students successfully overcome difficulties and accomplish the goals they want to achieve, they will be motivated to develop better CA (Bandura et al., 2001).

**FIGURE 1:**
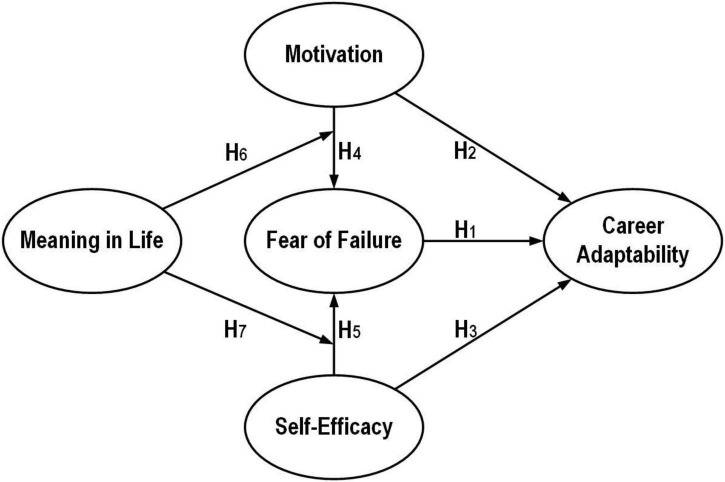
Research model.

Savickas (1997) proposed the career construction theory, which emphasizes the influence between an individual’s past experiences and social interactions, and gives cognitive meaning to these experiences through the individual’s subjectivity, thereby constructing their life course one step at a time (Johnston, 2018; Chen et al., 2020). The fear of failure can be interpreted as the cognitive interpretation that vocational students will not be able to achieve their desired goals in the future when they face their career events. The self-determination theory’s viewpoint emphasizes that each person makes decisions based on his or her own expectations (motivation) in whatever he or she does (Deci and Ryan, 2012; Ryan and Deci, 2017; Vasconcellos et al., 2020; Howard et al., 2021). Therefore, when the MT and SE of vocational students are high, it will positively correlate with their academic performance and social interaction in school and enhance their CA. Based on the above two theoretical and empirical studies, this study concluded that FF, MT, and SE are important factors affecting the CA of vocational students, and therefore the following hypotheses were developed (see Figure 1):

H_1_: FF will negatively affect CA

H_2_: MT will positively affect CA

H_3_: SE will positively affect CA

SDT suggests that human beings have three innate needs: Competence, Relatedness, and Autonomy, and that if these needs are met, it will bring about the best development and progress for the individual (Deci and Vansteenkiste, 2004; Deci and Ryan, 2012; Ryan and Deci, 2017). Conversely, when these needs are not met, the individual will act to change in response to the unmet needs (McDavid et al., 2014; Mouratidis et al., 2015; Howard et al., 2021). When they are unable to deal with the problem effectively, they can easily experience the negative fear of trying to avoid failure (Krijgsman et al., 2017; Weinstein and Ryan, 2011). Therefore, this study concluded that the stronger the MT of vocational students to achieve a specific goal, the higher the demand related to that goal, but when vocational students find out that they might not be able to achieve the goals they set, they would have a negative psychological state of FF (Choi, 2021). SE is the degree to which one believes in oneself and is able to use one’s abilities to do certain things and achieve certain goals (Bandura, 1994, 1997). In short, it is similar to a demonstration of self-confidence and the feeling of having enough ability to do the job; therefore, when the SE of vocational students is higher, the negative psychology of FF will be lower (Zhang et al., 2018; Urruzola and Bernaras, 2020; Rahimi and Hall, 2021). Therefore, the following hypotheses were proposed (see Figure 1):

H_4_: MT will positively affect FF

H_5_: SE will negatively affect FF

#### The moderating effect of meaning in life intervention

From the above, it is clear that ML is a subjective experience that people perceive as having value in their lives. This experience can make people passionate about life, motivated to achieve their goals, and willing to keep working until they achieve their desired goals (Steger et al., 2006; Li et al., 2018; Kıymaz, 2019). In other words, when vocational students decide to adjust their behavior in order to achieve their desired personal goals, they will go through a social cognitive process in which ML will have an impact on MT and SE. For vocational students who have realized (found) the meaning in their lives, their FF when facing problems or dilemmas is due to their psychological recognition that what they are about to accomplish is of value to them (Martela and Steger, 2016), and this recognition of the value of the goal can reduce the fear of failure that is only due to the increased fear of failure that comes with the motivation to succeed. This sense of identity also makes vocational students more open and fearless when facing the pressure of failure, reduces their uncertainty about the future, increases their sense of mastery over the future, enhances their SE, and reduces the influence of FF. On the contrary, if vocational students lack ML, they will be more concerned about the love and recognition brought by their external successful performance, so when they face failure, they will be more likely to retreat because of their weak sense of goal identification (Conroy et al., 2002; Sagar et al., 2007). In summary, the following hypotheses were developed for this study (see Figure 1):

H_6_ ML moderates the effect of MT on FF

H_7_ ML moderates the effect of SE on FF

### Research procedure

Based on the interest of this study, a model of CA was developed from the viewpoint of career construction theory and self-determination theory. A model of CA for vocational students was developed. This study uses data from the 2018 Programme for International Student Assessment (PISA), a global student assessment sponsored by the Organization for Economic Cooperation and Development (OECD). The target population is 15-16-year-old students and the content includes not only subject matter literacy (reading, math, science), but also some questionnaires. The aim of PISA is to understand the real-life literacy of students in a rapidly changing society and the education of socioeconomically disadvantaged students.

The program has been held every 3 years since 2000, and each year has a specific focus. The 2018 PISA focused on students’ “global competitiveness,” which includes students’ “adaptability,” which fits the focus of this study. Moreover, the questionnaire development and sampling process of PISA is rigorous and the analysis results will be close to the current situation of Taiwanese students. Therefore, this study was conducted using the 2018 PISA public data by adopting the secondary data method.

This study adopted the secondary data research method, and after a rigorous theoretical study and literature review, a research framework was formed. Five potential variables were then selected from the background variables in the 2018 PISA International Student Survey for the study (Career Adaptability, Fear of Failure, Motivation, Self-Efficacy, Meaning in Life). However, the variables in the PISA 2018 questionnaire were not originally designed for this study. Therefore, in order to be rigorous and to avoid over-interpretation by the researcher and misunderstanding by the reader, the definitions of the variables in this study were mostly extended based on the relevant concepts in the original design of the PISA components.

Finally, this study used the recent emergence of the least squares method for estimation and hypothesis validation through Smart PLS 3.3.7, using the CFA and SEM techniques. This method is increasingly used in marketing, organization management, human resource management, and information management (Hair et al., 2019; Khan et al., 2019; Sarstedt et al., 2020). The advantage of PLS-SEM over CB-SEM with maximum likelihood estimation is that the data can be distributed without constants, a smaller sample size can be used to compute complex models, and the mediated and adjusted models can be effectively estimated in one model (Joreskog, 1982; Hair et al., 2019; Shiau et al., 2019; Sarstedt et al., 2020; Cheah et al., 2021). Based on the above, this study selected the topics of interest from the PISA questionnaires according to the research objectives and used Smart PLS, one of the mainstream SEM analysis methods, to conduct the relevant validation.

### Measurements

#### Career adaptability

This study follows the PISA2018 definition of adaptability, which refers to the ability to adapt one’s thinking and behavior to the dominant cultural environment or new things, including the ability to understand multiple perspectives and the ability to overcome unfavorable circumstances (OECD, 2020a). This study adapted the Cognitive Flexibility scales for the PISA 2018 (Martin and Rubin, 1995; Dennis and Vander Wal, 2010). In addition to assessing vocational students’ adaptability in dealing with challenging or difficult situations, it also includes the ability to adapt to cross-cultural situations (OECD, 2020a). The CA questionnaire consists of six observation questions on a 5-point Likert scale (1 = *Very much like me*, 2 = *Mostly like me*, 3 = *Somewhat like me*, 4 = *Not much like me*, 5 = *Not at all like me*). The higher the value, the better the students’ cognitive adaptation ability. The Cronbach’s α of CA for this study was 0.92.

#### Fear of failure

FF can be seen as a way for students to assess their overall competence in the face of adversity (OECD, 2020b), where FF is defined as a tendency to avoid mistakes. The 2018 PISA adapted the Performance Failure Appraisal Inventory (PFAI) instrument (Conroy et al., 2002; Alkhazaleh and Mahasneh, 2016). From the feelings related to the fear of failure, such as, fear of experiencing shame and embarrassment or devaluing one self. one question was selected from each of the three dimensions and a total of three questions were selected to form a fear of failure questionnaire for vocational students. Using a 4-point Likert scale (1 = *Strongly disagree*, 2 = *Disagree*, 3 = *Agree*, 4 = *Strongly agree*), vocational students with higher response values were more afraid of failure than those with lower values. The Cronbach’s α of FF for this study was 0.89.

#### Motivation

Motivation is one of the reasons why students focus more on learning activities, and includes Perseverance, Openness to problem solving, Focus of control, and Intrinsic and instrumental motivation (OCED, 2013). The PISA 2018 motivation scale consists of four questions that asked students about their motivation to work and their motivation to achieve (OECD, 2020b). The 4-point Likert scale (1 = *Strongly disagree*, 2 = *Disagree*, 3 = *Agree*, 4 = *Strongly agree*) was used in this study, where the higher the response value, the higher the motivation of the vocational school students. The Cronbach’s α of MT for this study was 0.84.

#### Self-efficacy

SE is the degree to which an individual believes he or she is capable of participating in certain activities and completing specific tasks (Bandura, 1997). However, SE can be measured differently depending on the context, the person or the job. In other words, SE includes the ability to perform well-defined tasks (Bandura, 2006). In PISA 2018, SE focused on students’ academic SE (perceptions of their abilities), satisfaction with their knowledge and skills, and self-confidence (OECD, 2020b). There were five main topics, for example: “Proud of your achievements,” “Able to handle many things at once,” “Able to get through difficult situations by yourself,” “Finding solutions in difficult situations,” etc. A 4-point Likert scale (1 = *Strongly disagree*, 2 = *Disagree*, 3 = *Agree*, 4 = *Strongly agree*) was used in this study, and the higher the SE value, the better the SE of the vocational students. The Cronbach’s α of SE for this study was 0.81.

#### Meaning in life

ML means that people are able to transcend their short lives, believe that their lives have value, and recognize the purpose, goal, or mission of their lives (Steger et al., 2008; Steger, 2009). PISA 2018 defines the meaning of life as the extent to which 15-year-olds can understand or discover meaning in their lives (OECD, 2020b). It includes three questions on “having a clear meaning or purpose,” “discovering satisfying meaning,” and “what brings meaning to my life.” In this study, a 4-point Likert scale (1 = *Strongly disagree*, 2 = *Disagree*, 3 = *Agree*, 4 = *Strongly agree*) was used for ML, and the higher the response value, the better the student’s ML. The Cronbach’s α of ML for this study was 0.88.

## Results

Based on the interest of this study, a model of CA of vocational students was constructed from the viewpoint of career construction theory and self-determination theory. This study adopted a secondary data research method, from the perspective of data exploration, using the 2018 Programme for International Student Assessment (PISA) data. Partial Least Squares Structural Equation Modeling (PLS-SEM) was used for validation. The results are described as follows:

### Participants (demographic analysis of the main study)

Taiwan PISA 2018 surveyed a total of 2,659 questionnaires from 16-year-old students enrolled in three academic systems: senior high school, comprehensive high school, and vocational college. After eliminating the invalid and unfilled answers, a total of 2,377 (89.4%) valid data were used. Before the analysis, the data were checked and it was confirmed that the maximum and minimum values of each question were within the original scale, the skewness values of all questions ranged from -0.76 to 0.09, and the kurtosis values ranged from -0.43 to 2.16. The criterion of absolute value of skewness is less than 2 and absolute value of kurtosis is less than 7 for normal assignment, as suggested by Kline (2005). In other words, there were no filling errors in the data analyzed in this study and each question was consistent with the univariate norm.

From Table 1 below, we can see that among the 2,377 data, 1,026 comprehensive senior secondary students accounted for 43.2%, followed by 897 skill-based senior secondary students accounting for 37.7% and 454 five-year juniors. The least number of five-year junior college students, 454, accounted for 19.1%. The number of male and female students was 1,216 and 1,161, respectively, accounting for 51.2% and 48.4% of the total number of male and female students.

**TABLE 1:** Descriptive analysis (*N* = 2,377).

Background	*N*	%	Background	*N*	%
1. School Type			2. Gender		
(1) Skill-based senior secondary	897	37.7	(1) Female	1,216	51.2
(2) Comprehensive senior secondary	1,026	43.2	(2) Male	1,161	48.8
(3) Five-year junior college	454	19.1			

### Measurement model

This study followed a two-stage analysis (Anderson and Gerbing, 1988). First, the measurement model was validated to confirm that the questions measured well and that the constructs had convergent and discriminant validity. The data were then taken to the second stage of the structural model for hypothesis validation. The results of the analysis are explained as follows.

#### Construct reliability and validity analysis

In the measurement mode stage, a confirmatory factor analysis (CFA) was conducted with reference to the ideal measurement model numerical criteria suggested by scholars Fornell and Larcker (1981) and Hair et al. (2009): (1) items’ standardized factor loadings were greater than 0.6; (2) component reliability was greater than 0.7; and (3) mean variance extraction was greater than 0.5. The results of the measurement mode in this study included the following modulations in order: Fear of Failure (FF) three questions; Adaptability (AP) six questions; Motivation (MT) four questions; Self-Efficacy (SE) five questions; and Meaning in Life (ML) three questions, giving a total of five sections and 21 questions. All 21 questions had Factor Loadings ranging from 0.64 to 0.93, and the five constructs had CR values ranging from 0.87 to 0.93, and AVE values ranging from 0.57 to 0.81. These values are in line with the criteria suggested by scholars. See Table 2 below for details.

**TABLE 2:** Construct reliability and validity analysis.

Variable item		FL	CR	AVE
**1. Career Adaptability**				
CA1	I can deal with unusual situations.	0.83	0.93	0.70
CA2	I can change my behavior to meet the needs of new situations.	0.83		
CA3	I can adapt to different situations even when under stress or pressure.	0.86		
CA4	I can adapt easily to a new culture.	0.85		
CA5	When encountering difficult situations with other people, I can think of a way to resolve the situation.	0.88		
CA6	I am capable of overcoming my difficulties in interacting with people from other cultures.	0.79		
**2. Fear of Failure**				
FF1	When I am failing, I worry about what others think of me.	0.90	0.93	0.81
FF2	When I am failing, I am afraid that I might not have enough talent.	0.93		
FF3	When I am failing, this makes me doubt my plans for the future.	0.88		
**3. Motivation**				
MT1	I find satisfaction in working as hard as I can.	0.82	0.90	0.68
MT2	Once I start a task, I persist until it is finished.	0.85		
MT3	Part of the enjoyment I get from doing things is when I improve on my past performance.	0.87		
MT4	If I am not good at something, I would rather keep struggling to master it than move on to something I may be good at.	0.76		
**4.Self-Efficacy**				
SE1	I usually manage one way or another.	0.70	0.87	0.57
SE2	I feel proud that I have accomplished things.	0.64		
SE3	I feel that I can handle many things at a time.	0.75		
SE4	My belief in myself gets me through hard times.	0.83		
SE5	When I’m in a difficult situation, I can usually find my way out of it.	0.84		
**5.ML**				
ML1	My life has clear meaning or purpose.	0.84	0.92	0.80
ML2	I have discovered a satisfactory meaning in life.	0.93		
ML3	I have a clear sense of what gives meaning to my life.	0.90		

#### Construct discriminant validity

According to the results of discriminant validity analysis in Table 3 below, the AVE method was used in this study. Fornell and Larcker (1981) proposed that for the average variance extracted (AVE) of each construct, when the root number is greater than the correlation coefficient of each construct, it means that each construct has discriminant validity. As shown in Table 3 below, the diagonal lines between the surfaces are AVE open root values with a minimum value of 0.76, which is greater than the correlation between the surfaces (−0.10 ∼0.44). This shows that the constructs of this study have good discriminant validity. In summary, the data measurement model of this study is good and the results meet the criteria suggested by academic experts and are suitable for subsequent overall model and hypothesis validation.

**TABLE 3:** Construct discriminant analysis & variance inflation factor.

	AVE	1.CA	2.FF	3.MT	4.SE	5.ML
1.Career Adaptability	0.70	***0.84***	*1*.*06*	*1.26*	*1.22*	
2.Fear of Failure	0.81	–0.03	***0.90***			*1.31*
3.Motivation	0.68	0.32	0.19	***0.83***		*1.24*
4.Self-Efficacy	0.57	0.41	–0.05	0.40	***0.76***	*1.40*
5.Meaning in Life	0.80	0.24	–0.10	0.34	0.44	***0.89***

The figures in bold and italics in the diagonal direction represent the square roots of AVEs; the off-diagonal elements are the correlation estimates; the upper triangular matrix are inner VIF values.

### Structural model

The standardized root mean square residual (SRMR) of this study was 0.05, which is smaller than the SRMR value of 0.08 suggested by Hu and Bentler (1999); therefore, the model of this study has good model fit. To be rigorous, the Variance Inflation Factor (VIF) was checked before conducting the hypotheses validation and the VIF values of each configuration ranged from 1.06 to 1.40, all of which were less than 3.3, which is in line with the VIF value of 3.3 proposed by Kock and Lynn (2012). In other words, there is no co-linearity among the variables of the components, making it suitable for regression analysis.

#### Path analysis

**FIGURE 2:**
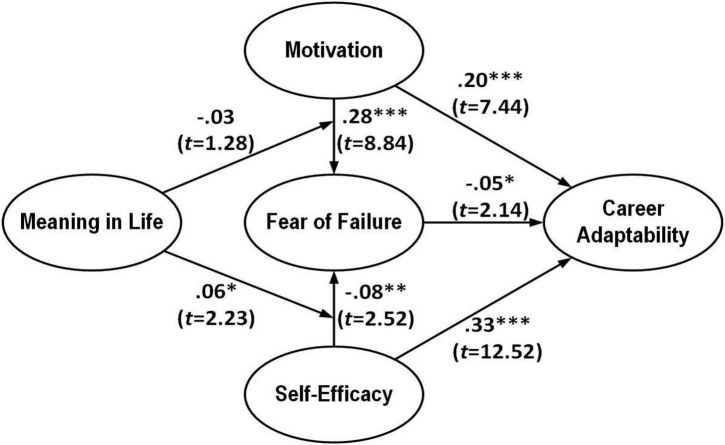
Validation of the research model. ^∗^*p* < 0.05, ^∗∗^*p* < 0.01, ^∗∗∗^*p* < 0.001.

In the overall model, based on the research objectives and previous literature, the main effects of the four pathways between “fear of failure,” “career adaptability,” “motivation,” and “self-efficacy” were first tested for hypotheses H1∼H5. As shown in Table 4 and Figure 2, FF significantly and negatively affected CA, supporting H_1_ (FF→CA: β = −0.05, *t*-value = 2.14, [−0.10, −0.00]). MT had a significant positive impact on CA and FF, supporting H_2_ (MT→CA: β = 0.20, *t*-value = 7.44, [0.14, 0.25]) and H_4_ (MT→FF: β = 0.28, *t*-value = 8.84, [0.21, 0.34]). SE positively and significantly affected CA, supporting the establishment of H_3_ (SE→CP: β = 0.33, *t*-value = 12.52, [0.27, 0.38]). Finally, SE negatively and significantly affected FF and so supported H_5_ (SE→FF: β = −0.08, *t*-value = 2.52, [−0.14, −0.02]).

**TABLE 4:** Research hypothesis verification.

Hypotheses				Point estimate				Bias-corrected CI 95%		Result
Variable relationship				Path coefficient	Standard deviation	*t* value	*P*-value	2.50%	97.50%	
H_1_	FF	→	CA	−0.05	0.02	2.14	*	–0.10	–0.00	**Accept**
H_2_	MT	→	CA	0.20	0.03	7.44	***	0.14	0.25	**Accept**
H_3_	SE	→	CA	0.33	0.03	12.52	***	0.27	0.38	**Accept**
H_4_	MT	→	FF	0.28	0.03	8.84	***	0.21	0.34	**Accept**
H_5_	SE	→	FF	-0.08	0.03	2.52	**	–0.14	–0.02	**Accept**
H_6_	ML × MT	→	FF	-0.03	0.03	1.28	0.20	–0.09	0.02	**False**
H_7_	ML × SE	→	FF	0.06	0.03	2.23	*	0.01	0.11	**Accept**

FF Fear of Failure; CA Career Adaptability; MT Motivation; SE Self-Efficacy; ML Meaning in Life; Bootstrap 5,000 times.

*p < 0.05, **p < 0.01, ***p < 0.001.

#### Moderate effect

**FIGURE 3:**
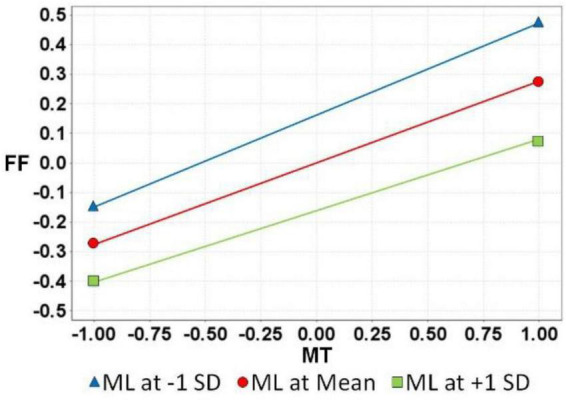
H6 Moderating effect.

**FIGURE 4:**
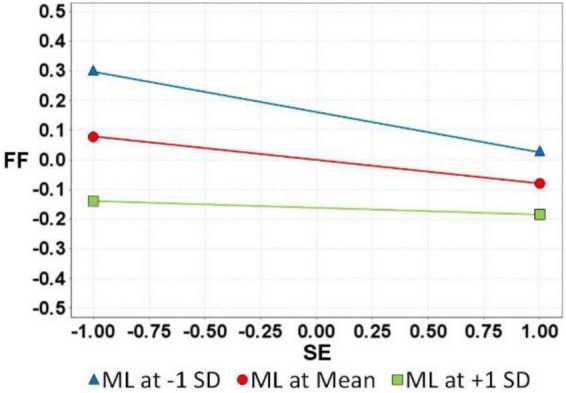
H7 Moderating effect.

After the verification of H_1_∼H_5_, the ML moderation effect of H_6_ and H_7_ was verified; the results are as follows: (see Table 4 and Figure 2) CA on MT and FF showed no moderation effect. Therefore, H6 was not supported (ML × MT→FF: β = −0.03, *t*-value = 1.28, [−0.09, 0.02]). CA had a positive moderating effect on FF and SE. Therefore, H_7_ was supported (ML × SE→FF: β = 0.06, *t*-value = 2.23, [0.01, 0.11]). Figures 3, 4 below show the moderation effect of ML.

### Discussion

This study combined career construction theory (Savickas, 1997) and self-determination theory (Deci and Ryan, 2002, 2012; Ryan and Deci, 2017) to explore the effect of vocational students’ motivation, self-efficacy, fear of failure, and meaning in life on their career adaptability. The findings are discussed below.

First, this study found that FF did have a negative effect on vocational students’ CA. Although few empirical studies have been conducted to investigate the direct relationship between the two, vocational students are prone to self-limiting behaviors in order to avoid the psychological pressure of failure, which thus reduces the chance of career adaptability development (Elliot and Thrash, 2004; Vaughn et al., 2021). Vocational students’ MT had a positive impact on CA (Fang et al., 2018; Schuesslbauer et al., 2018). Past research has shown that whether the source of inner motivation, outter motivation can contribute to successful learning experiences for vocational students, which in turn has a positive impact on their CA and careers (Brooks et al., 2012; Davis and McPartland, 2012; Schuesslbauer et al., 2018). Finally, SE triggers the ability of vocational students to act on what they are doing (Bandura, 2006). This is similar to the career decision-making self-efficacy or job search self-efficacy mentioned in previous studies. The essence is the same: to give people the confidence to overcome all the difficulties they may encounter in their careers and to develop better CA (Bandura et al., 2001; Guan et al., 2013; Guan et al., 2016).

Second, this study found that the higher the vocational students’ MT, the more afraid they were of failure. This phenomenon is similar to the previous studies in which students’ motivation was based on external factors, such as to achieve the requirements of school teachers, parents’ expectations and to achieve external performance; such MT with clear achievement goals and performance orientation is indeed closely related to students’ FF (Conroy and Elliot, 2004; Sagar and Lavallee, 2010; Krijgsman et al., 2017; Giel et al., 2020; Taylor et al., 2021).

The SE of vocational students negatively affects their FF. When people know that they can effectively handle many demanding events (self-efficacy), it reduces their FF negatively (Weinstein and Ryan, 2011; Krijgsman et al., 2017). This is similar to previous research on the effects of SE in health profession undergraduate students to overcome FF and reduce academic procrastination, and SE in music students to reduce anxiety when performing music (Zhang et al., 2018; Urruzola and Bernaras, 2020).

Finally, this study found that ML positively moderated the negative effect of SE on FF among vocational students, but there were no statistically significant differences in how MT affected FF. Overall, scholars have suggested that ML can make people passionate about life, motivated to achieve goals, and willing to keep working until they achieve their desired goals (Steger et al., 2006; Li et al., 2018; Kıymaz, 2019). The above conclusions seem to be slightly inconsistent with the results of the present study. However, when explored further, ML is something that makes people focus on the intrinsic value of their lives and the desire to have a life worth living, and creates a commitment to a career (Martela and Steger, 2016; Li et al., 2018). From this point of view, ML should be adjusted to reduce the influence of MT on the FF of vocational students. However, the results obtained in this study are that ML does not moderate the effect of MT on FF. The main reason for this result may be that ML is obtained through a process of self-discovery, which is a transformation of one’s internal mental journey. However, the MT of the measurement in this study is more of an external instrumental motivation, and it would be relatively difficult to get an internal factor to interact with an external influence, thus producing a statistically insignificant moderation effect. On the contrary, SE itself is an inner belief that produces the ML moderation effect of SE on FF.

## Conclusion

### Theoretical implications

Savickas’ (1997) career construction theory emphasized the importance of CA and stressed that CA is constructed from an individual’s past experiences and subjective perceptions. Self-determination theory is the key behind each person’s behavior. Therefore, everything that each person does is determined by his or her own internal expectations (Deci and Ryan, 2012; Ryan and Deci, 2017). In addition to the four aspects of career focus, career control, career curiosity, and career confidence in the original CCT theory (Savickas, 1997, 2005), this study also added MT, SE, and FF in response to the satisfaction of internal needs in the SDT theory. The three elements of FF, MT, and SE were used to supplement the effects on CA. In particular, this study investigated 16-year-old students in the technical vocational system who were in their first year of streaming into the technical vocational system and were facing the uncertainty of entering technical vocational education. Therefore, if vocational students have good CA, it will not only have an impact on their current learning in school, but may also lead to a better socio-economic status or better quality of life after graduation, which will affect them throughout their life.

### Practical implications

Nowadays, our environment has changed drastically, especially in the past few years due to the impact of COVID-19 and digitalization. Since CA is a key ability that affects the future career development of vocational students, and the process of constructing CA is very diverse and encompasses a wide range of aspects, this study highlights the importance of paying attention to vocational students’ CA. The following are recommendations for technical vocational education practices based on the findings of this study.

First, vocational students’ FF negatively affects their CA, while MT and SE positively affect CA. While it is true that FF may adversely affect student performance, the relative FF may also be a disguised incentive for some students to improve their skill acquisition or to develop other strategies for academic performance outside of academics (Nsiah, 2017; Alabduljabbar et al., 2022). Therefore, for 16-year-old vocational school students, the negative effect of FF on CA may not be as great as expected due to the interaction of MT and SE.

Secondly, MT is like a double-edged sword, as it increases CA but also increases FF. Conversely, SE can improve vocational students’ CA and reduce their FF. Therefore, the development of SE and self-confidence should be prioritized over the enhancement of MT when applied to the CA enhancement strategies of vocational students. In the data of this study, it was found that the loading value of SE2 “I feel proud that I have accomplished things”, the loading value of this question is low, Therefore, it is urgent to cultivate a sense of accomplishment among 16-year-old vocational school students. The source of power for future success is to create a positive learning environment or internship field in school, so that vocational students can gain a sense of accomplishment and become confident, willing to help others, and can realize their self-worth.

Finally, since ML can positively moderate the negative effect of SE on FF, in other words, in order to reduce vocational students’ FF, besides cultivating SE and confidence, perhaps we can also start from life education-related courses to make them understand the meaning of their own lives. This is a subjective feeling that can only be realized by individuals (in this case, vocational students) after they have gone there themselves and found their own value in life. Therefore, in the design of the curriculum, it is necessary to provide students with more opportunities to explore ML through more diversified life experiences and a curriculum design that is closer to their lives.

### Limitations and future study

The study was conducted in accordance with CCT and SDT, and some interesting results were found. However, there are still relevant limitations as follows. First, because the culture, social environment, and values of each country are different, the results may not be the same when extrapolated to different countries. Second, CA and the ML will vary from generation to generation depending on the time and space in which they live. Finally, the present study can be used as the basis for further research, which can be extended to students of different age groups or academic systems, or further explored for vocational students of different countries, cross-cultures, or different generations.

## Data availability statement

Publicly available datasets were analyzed in this study. This data can be found here: https://www.oecd.org/pisa/data/2018database.

## Ethics statement

Ethical review and approval was not required for the study on human participants in accordance with the local legislation and institutional requirements. Written informed consent from the patients/participants or patients/participants legal guardian/next of kin was not required to participate in this study in accordance with the national legislation and the institutional requirements.

## Author contributions

All authors listed have made a substantial, direct, and intellectual contribution to the work, and approved it for publication.
